# Some Salt with Your Statin, Professor?

**DOI:** 10.1371/journal.pbio.1001768

**Published:** 2014-01-21

**Authors:** Malcolm Macleod

**Affiliations:** Centre for Clinical Brain Sciences, University of Edinburgh, Edinburgh, United Kingdom

## Abstract

We know that clinical trials sponsored by the pharmaceutical industry are likely to exaggerate benefit and minimise harms. But do these biases extend to their sponsorship of non-human animal research? Using systematic review and meta-analysis Bero and colleagues show that, in the case of statins, things are a little more complicated. While the conclusions of industry-sponsored studies were indeed more enthusiastic than warranted by their data, the data themselves painted a picture more conservative than was seen in non-industry-sponsored studies. This behaviour is consistent with maximising the return on investment, seeking robust data before embarking on a clinical trial, and, once that investment has been made, making every effort to “prove” that the drug is safe and effective if this is at all credible. The findings suggest that there is something different about industry-sponsored non-human animal research, perhaps reflecting higher standards than is the case elsewhere. Perhaps the academic community can learn something from our colleagues in the commercial sector.

## Abstract 2

In this primer, Malcolm Macleod outlines the use of meta-analysis to assess the validity of the outcome reported for preclinical studies and explores the reasons why Lisa Bero and colleagues find that industry-funded studies show smaller effect sizes than non-industry-funded studies do.

It is now pretty clear that, in clinical trials, sponsorship from the pharmaceutical industry is associated with substantial and important overstatement of how effective drugs are, and with understatement of adverse effects [1]. Of course, these are average effects, and so are insufficient to label the whole industry bad. Nonetheless, there are many examples where industry has been shown to seek to subvert rational interpretation of trial data to influence guideline development and prescribing behaviour [2]–[4]. These examples lead to the reasonable conclusion that findings from trials sponsored by the pharmaceutical industry need to taken with more salt than is probably good for you.

What then of other research used to inform the drug development process? What of the in vitro and in vivo non-human research supported by industry, either in companies' own laboratories or that companies fund in contract research organisations or in academic collaborations? Are the findings of such studies credible? And how do those findings compare with “proper” research conducted by dispassionate academics?

These are important questions, but how could we find this stuff out? In the same way that it would be difficult to conduct a randomised controlled trial of the effect of living in Scotland on your chance of having a stroke, it is difficult to do an experiment to test whether the funding source for a study influences the outcome. We have to rely on observational (rather than experimental) research, and we need to be much more cautious in our approach and in our conclusions.

Over the last few years there has been a big increase in the use of such an observational approach to better understand the strengths and weaknesses of different research domains. The Cochrane Collaboration began as an attempt to give reliable summaries of the effectiveness of treatments in human clinical trials [5], but along the way the data collected have also allowed investigation of whether studies with certain characteristics tended to give overstatement or understatement of these summary treatment effects [6]. The insights arising from this approach, and the improvements in trial design that they have driven, are just as important as the improved information to guide treatment decisions. This approach has been used by others—notably Lisa Bero, the senior author of the research article presented here—in a series of important papers that identified the prevalence and impact of funding bias in human research [7],[8].

Those wishing to study, and to improve, other research domains such as non-human animal research have been able shamelessly to borrow from the experience of the Cochrane Collaboration. Using a systematic approach to data retrieval we can assemble an unbiased cohort of relevant studies, then observe associations between different aspects of experimental design and the magnitude of the effects reported. What we're looking for are design features that are consistently associated with either under- or overestimation of biological effects.

Of course, meta-analyses of clinical trial data put together a small number of large studies measuring a common treatment effect, whereas in animal studies there is usually a large number of small studies measuring different effects (dose, stage of illness, different animals), which means the approach used has to be adjusted slightly, but still, the approach has been fruitful.

For a large number of non-human animal disease models, studies at risk of bias (for example, those without randomisation or blinding) give larger estimates of treatment effects [9]–[13]; the majority of studies are at risk of bias [9]–[14]; and journal impact factor is no guarantee of low risk of bias [15]. These findings influenced the development of reporting standards for stroke [16] and non-human animal research more generally [17],[18], and these are beginning to make an impact.

One difficulty in using meta-analysis is in working out how to combine different outcome measures, often from different animals. A 0.1-mm increase in aortic arch atheroma is probably less important in a Scot than it is in a mouse, so we need to transform data onto a common scale. In standardised mean difference (SMD) meta-analysis, the effect is standardised to the observed variance [19]. Because—in large studies at least—this variance is a property of the biology being studied rather than of the scale being used, it allows effects to be converted to a common scale. So, by way of an example: in 2012 the variance of the monthly average temperature across 258 weather stations in California was 12.55°F, or 6.98°C—from which we can calculate that 1°C is the same as 1.80°F, or 0.14 standardised units, and so we have a common scale.

While this approach is very useful in clinical meta-analyses (where the large number of participants in each group allows a precise estimate of the population variance), it becomes less useful where group size is small, because here the observed variance is a less precise estimate of the population variance. This introduces a measurement error to the conversion between different scales.

Further, this observed variance represents a combination of underlying biological variation in the phenomena being measured and of variation arising from measurement error and from the way the experiment was performed. Experiments with low measurement error and good protocol compliance will therefore have lower aggregate variance than those with high measurement error and poor protocol compliance. Since the variance is the denominator in the calculation of the size of differences between groups, any given effect size will be artificially larger in studies with low measurement error and experimental variability.

The demonstration that experiments with low methodological quality can give inflated estimates of treatments effects, and that most experiments appear to be of low methodological quality, leads to the question of who might be the worst offenders. Since clinical trials sponsored by the pharmaceutical industry seem to be at greater risk of bias than others, a lazy assumption might be that their non-human animal research is similarly confounded, as they seek to rush compounds to market to maximise profitability.

However, a few straws in the wind hint this might not be the case. One way companies identify drug targets is by reading what's out there in the literature and, if something looks interesting, seeking to replicate the findings. Bayer scientists found inconsistencies in 43 of 65 studies when they tried to replicate them in-house [20]. Scientists in the haematology and oncology departments at Amgen were able to replicate findings in only six out of 53 publications identified as “landmark” studies [21]. When the ALS Therapy Development Institute tried to replicate published findings of drug efficacy in the superoxide dismutase mouse model of motor neuron disease (amyotrophic lateral sclerosis), not one of seven interventions retained efficacy [22]. Implementation of good laboratory practice standards is much more advanced in industry labs, and for some types of experiments these standards are a legal requirement. Indeed, a scientific researcher was recently jailed in Scotland for research fraud [23]. So, could it be that industry-sponsored research is actually more rigorous than academic research?

Taking the example of statin treatments for atheroma, David Krauth, Andrew Anglemyer, Rose Philipps, and Lisa Bero address this issue head-on [24]. Using systematic review they identified non-human animal studies describing the efficacy of statins. Their methodology is secure, with an a priori analysis plan, clear inclusion and exclusion criteria, and duplicate extraction of key variables from identified publications. They found low levels of reporting of measures known to reduce the risk of bias, with blinded assessment of outcome reported in only 22 of 49 studies, and no studies reporting full randomisation or a sample size calculation. Reassuringly, the quality of reporting seems to have improved somewhat since publication of the ARRIVE guidelines in 2010. However, there is still clearly a long way to go.

On the question of the influence of the study sponsor, Bero and colleagues identified 19 studies sponsored in whole or part by industry, 28 sponsored by non-industry sources, and 16 with no statement of sponsorship or a statement of no sponsorship. Focussing on those studies where sponsorship status was known, they found that the results of nine of 19 industry-sponsored studies (43%) and 18 of 28 non-industry-sponsored studies (72%) supported the efficacy of statins. This finding was confirmed in a subset of 38 studies with sufficient data to allow meta-analysis; statins were reported to improve outcome by 0.73 SMD units in industry-sponsored studies, while in studies with other sponsorship the improvement was 1.99 SMD units. This difference is highly significant—I calculate an excess of efficacy in non-industry-sponsored studies of 173% (95% confidence interval 52% to 293%). Put simply, studies with non-industry sponsorship report that statins are almost three times more effective than do industry-sponsored studies.

As interesting, however, is the analysis of the interpretation placed on the findings in each of the included studies. Of 19 industry-sponsored studies, the conclusion of 18 favoured the use of statins (95%), while of 28 non-industry-sponsored studies, only 21 did so (75%). This is striking for two reasons: first, in both cohorts the conclusion appears to be more enthusiastic than the findings presented, and second, this phenomenon appears to be much more marked in studies with industry sponsorship.

So what's going on? Of course, these observed differences may be due to some other, unmeasured difference between the contributing studies, but the analyses were prespecified and such a confound appears unlikely. If industry-sponsored studies were of consistently larger variance, then the effect sizes observed would appear smaller in SMD units, but there is no reason to suspect that this was the case here.

It does therefore appear that findings from research sponsored by industry are more conservative than those sponsored by non-industry sources, but the interpretation of those data is, in contrast, less conservative. Why might this be?

In a series of univariate analyses the authors examined the impact of three factors—randomisation, blinding, and accounting for all animals—that might increase the risk of bias. Even when these were taken into account, non-industry-sponsored studies gave significantly higher estimates of efficacy, implying that some other factors were responsible. This might happen if “randomisation” and “blinding” meant different things in industry-sponsored studies, or through the impact of some other, unmeasured risk of bias, or through some gestalt of industry-sponsored studies that is not described by the variables tested. Alternatively, academic studies exploring pathophysiology might chose circumstances that maximise the observed effect size, to give greater statistical power to experiments testing inhibition of those effects.

In my view it is likely that the impact of approaches to research management and the regulatory environment that apply to some parts of industry—particularly standards for internal reporting—extends to most of the non-human animal research activity with which they are involved, whether or not it is performed in-house. That is, non-human animal work sponsored by industry is likely to be performed and reported to a higher quality, and to be at lower risk of bias, than work sponsored by others. This would explain the difficulty industry has in replicating the results of research conducted in academic labs. However, the interpretation, or “spin”, with which industry-sponsored work is presented does appear to be an issue, with exaggeration of the conclusions to favour the drug being tested.

This makes sense—for industry there is a clear financial interest in being absolutely secure in the non-human animal data for a compound before embarking on a clinical trial, so there is a real motivation to get the preclinical data as good as they can be. Clinical trials are expensive, and so it is worth investing much time and effort, and perhaps even funding multicentre “phase 3” animal studies [25]–[27], to maximise the prospects for success. But when that money has been spent (and for statins it largely has been), the motivation is to present an analysis of the available data that is most supportive for clinical use. So, if a drug is a turkey, try to find that out before spending a fortune taking it to clinical trial—and if it's too late for that, try to convince everyone that the non-human animal and clinical trial data supporting an efficacy for *Meleagris gallopavo* (commonly known as the wild turkey) are more convincing than they might at first appear.

In contrast, academic researchers are rewarded not for the marathon but for the sprint—for a high-impact publication describing a part of the jigsaw, not for the body of work that shows the whole picture. To them, substantial efficacy in a single study is, in some respects, an end rather than a beginning.

Bero and colleagues have made an important contribution; their findings suggest that academic researchers might learn good practice in the management, conduct, and reporting of non-human animal research from colleagues in industry, and reinforces the importance for readers of research reports to focus on methods and data rather than on abstracts and conclusions.

## Abbreviations

SMD: standardised mean difference
